# Extended Synaptotagmins 1 and 2 Are Required for Store-Operated Calcium Entry, Cell Migration and Viability in Breast Cancer Cells

**DOI:** 10.3390/cancers16142518

**Published:** 2024-07-11

**Authors:** Pedro C. Redondo, Jose J. Lopez, Sandra Alvarado, Isaac Jardin, Joel Nieto-Felipe, Alvaro Macias-Diaz, Vanesa Jimenez-Velarde, Gines M. Salido, Juan A. Rosado

**Affiliations:** Department of Physiology, Institute of Molecular Pathology Biomarkers, University of Extremadura, 10003 Caceres, Spain; jjlopez@unex.es (J.J.L.); sandraam@unex.es (S.A.); ijp@unex.es (I.J.); joelnf@unex.es (J.N.-F.); alvaromd@unex.es (A.M.-D.); vjimenezpw@alumnos.unex.es (V.J.-V.); gsalido@unex.es (G.M.S.)

**Keywords:** E-Syt1, E-Syt2, SOCE, cell migration, cell viability

## Abstract

**Simple Summary:**

Breast cancer is one of the most common types of cancer in women. Breast tumors can be grouped in, at least, three different molecular subtypes, including luminal, HER2+ and triple negative subtypes. Store-operated Ca^2+^ entry (SOCE) is a mechanism for Ca^2+^ influx that supports a variety of cancer hallmarks in breast cancer cells. The key molecular players of SOCE are the endoplasmic reticulum Ca^2+^ sensor STIM1 and the highly Ca^2+^-selective Orai1 channels in the plasma membrane. The aim of the present study is to elucidate the functional role of extended synaptotagmins 1 and 2 in the activation of SOCE by facilitating the tethering of the plasma membrane and the membrane of the endoplasmic reticulum. Our results indicate that extended synaptotagmins 1 and 2 are required for SOCE, migration and viability in luminal MCF7 and triple negative breast cancer cells, without having any effect on SOCE in non-tumoral breast epithelial cells.

**Abstract:**

Extended synaptotagmins (E-Syts) are endoplasmic reticulum (ER)-associated proteins that facilitate the tethering of the ER to the plasma membrane (PM), participating in lipid transfer between the membranes and supporting the Orai1–STIM1 interaction at ER–PM junctions. Orai1 and STIM1 are the core proteins of store-operated Ca^2+^ entry (SOCE), a major mechanism for Ca^2+^ influx that regulates a variety of cellular functions. Aberrant modulation of SOCE in cells from different types of cancer has been reported to underlie the development of several tumoral features. Here we show that estrogen receptor-positive (ER+) breast cancer MCF7 and T47D cells and triple-negative breast cancer (TNBC) MDA-MB-231 cells overexpress E-Syt1 and E-Syt2 at the protein level; the latter is also overexpressed in the TNBC BT20 cell line. E-Syt1 and E-Syt2 knockdown was without effect on SOCE in non-tumoral MCF10A breast epithelial cells and ER+ T47D breast cancer cells; however, SOCE was significantly attenuated in ER+ MCF7 cells and TNBC MDA-MB-231 and BT20 cells upon transfection with siRNA E-Syt1 or E-Syt2. Consistent with this, E-Syt1 and E-Syt2 knockdown significantly reduced cell migration and viability in ER+ MCF7 cells and the TNBC cells investigated. To summarize, E-Syt1 and E-Syt2 play a relevant functional role in breast cancer cells.

## 1. Introduction

Intracellular Ca^2+^ signaling is a key player in the modulation of cell function upon agonist stimulation. Among the mechanisms involved in intracellular Ca^2+^ homeostasis, store-operated Ca^2+^ entry (SOCE), a process controlled by the filling state of the intracellular Ca^2+^ stores, plays a relevant functional role supporting a number of cellular events including regenerative Ca^2+^ oscillations [1], immune cell activation [2] or lactation [3]. Depletion of the intracellular agonist-releasable Ca^2+^ stores, especially the endoplasmic reticulum (ER), leads to the activation of the Orai/CRAC (Ca^2+^ release activated Ca^2+^) channels through the ER-resident stromal interaction molecules (STIM1 and/or STIM2). STIM proteins sense intraluminal ER Ca^2+^ levels and communicate the filling state of the Ca^2+^ stores to the CRAC channels. Orai1 is the canonical pore-forming subunit of the CRAC channels that conducts the highly Ca^2+^-selective current *I*_CRAC_ [4,5,6,7,8,9,10].

The modulation of Ca^2+^ entry across the plasma membrane through CRAC channels is crucial to generate physiological Ca^2+^ signals stimulated by agonists as well as for intracellular Ca^2+^ homeostasis. The regulation of SOCE involves from the heteromerization of the mammalian Orai isoforms, Orai1, Orai2 and Orai3, and STIM homologs, STIM1 and STIM2 [11,12], to the participation of different regulatory proteins, including SARAF (SOCE-associated regulatory factor), which is involved in slow Ca^2+^-dependent inactivation of CRAC channels [13], STIMATE, CRACR2A (CRAC regulator 2A), septins, golli and ORMDL3 [14]. Among the proteins supporting SOCE, extended synaptotagmins (E-Syts) are ER-associated proteins that facilitate the tethering of the ER to the PM. Three E-Syts have been identified in mammalian cells, E-Syt1, E-Syt2 and E-Syt3. E-Syts consists of a short N-terminal domain, a single transmembrane domain and a C-terminal region that contains a synaptic mitochondrial lipid-binding protein (SMP) domain and either five (E-Syt1) or three (E-Syt2 and E-Syt3) C2 domains, involved in Ca^2+^- and phospholipid-binding, as well as in protein–protein interaction [15]. E-Syts participate in Ca^2+^ and lipid signaling. For instance, the formation of E-Syt-dependent ER–PM junctions upon cell stimulation with agonists that hydrolyze PtdIns(4,5)P_2_ and increase cytosolic Ca^2+^ concentration has been shown to reverse diacylglycerol accumulation in the PM by transferring it to the ER for recycling [16]. Furthermore, E-Syt1 participates in the regulation of glutamate receptor trafficking and synaptic plasticity [17]. Concerning the STIM–Orai interaction, the short isoform of E-Syt2 has been shown to recruit STIM1 at ER–PM junctions via a direct interaction [18]. In addition, at resting conditions, STIM2 clusters at ER–PM junctions recruit Orai1 to regulate basal Ca^2+^ entry, a process that requires E-Syt2/3 and the juxtaposition of IP_3_R [19]. E-Syt1 has been reported to mediate the formation of ER–PM junctions that support Orai1–STIM1 interaction [20].

Cancer cells are featured by uncontrolled proliferation, evasion of apoptosis and invasion of local tissue [21]. Among the cellular mechanisms underlying these changes, abnormal Ca^2+^ homeostasis due to remodeling of the expression and/or function of Ca^2+^ handling proteins, including the SOCE core proteins STIM1 and Orai1, plays a relevant role. Breast cancer cells of the different subtypes exhibit a significant remodeling of Orai proteins and are strongly dependent on the function of specific Orai channels. For instance, Orai1 expression is enhanced in the estrogen receptor-positive (ER+) and triple-negative subtypes [22,23], and both triple-negative breast cancer (TNBC) differentiated cells and stem cells are remarkably dependent on Orai1 expression and function [24]. Furthermore, Orai3 plays a major role in the activation of SOCE in ER+ breast cancer cells, which is important for Ca^2+^ homeostasis [25,26].

In the present study, we have investigated the functional role of E-Syt1 and E-Syt2 in SOCE in non-tumoral breast epithelial cells as well as in ER+ and TNBC cells. Our results indicate that E-Syt1 and E-Syt2 are overexpressed at the protein level in most of the ER+ and TNBC breast cancer cell lines investigated as compared to that in non-tumoral breast epithelial MCF10A cells. In addition, E-Syt1 and E-Syt2 play a functional role in the activation of SOCE, cell migration and viability in ER+ MCF7 cells and the TNBC cell lines MDA-MB-231 and BT20.

## 2. Results

### 2.1. E-Syt Expression in Breast Cancer and Non-Tumoral Breast Epithelial Cells 

**Figure 1:**
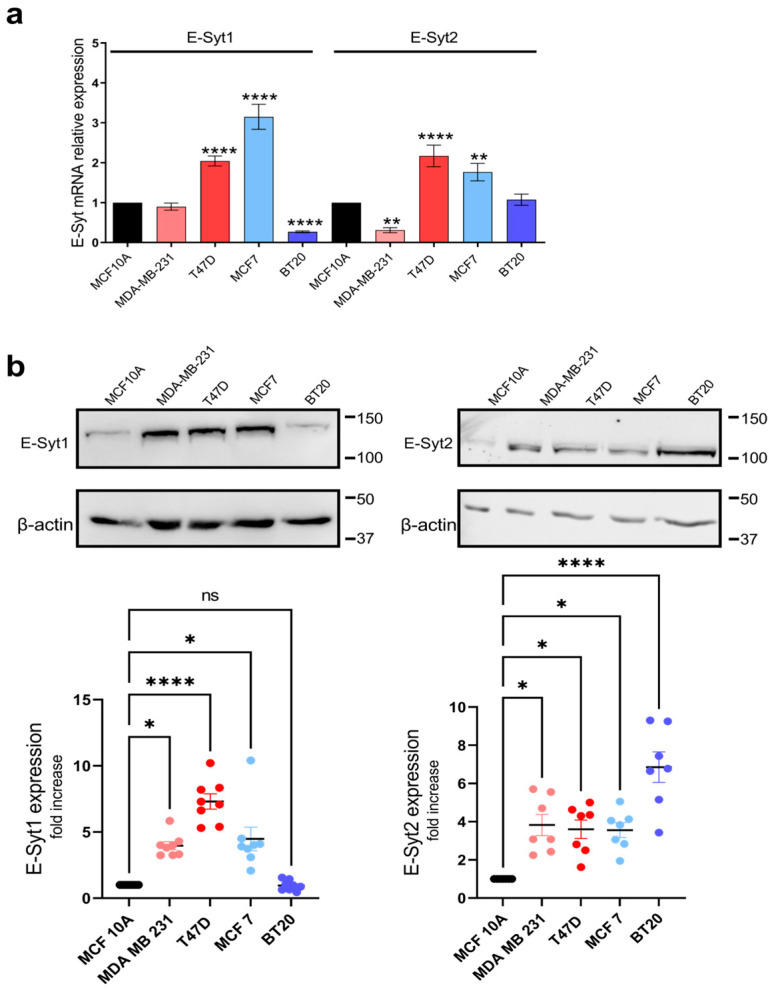
E-Syt mRNA and protein expression in breast cancer cell lines. (**a**) RT-qPCR expression analysis of E-Syt1 and E-Syt2 mRNA transcripts in the non-tumoral MCF10A cell line, the ER+ MCF7 and T47D breast cancer cell lines and triple-negative MDA-MB-231 and BT20 breast cancer cell lines. Values were normalized to GAPDH expression and represented as mean expression relative to MCF10A ± S.E.M.; *n* = 4. Data were statistically analyzed using the Kruskal–Wallis test with multiple comparisons (Dunn’s test) (** *p* < 0.01 and **** *p* < 0.0001 as compared to MCF10A cells). (**b**) Cells from the non-tumoral MCF10A cell line, as well as the ER+ MCF7 and T47D cells and TNBC MDA-MB-231 and BT20 cells, were lysed. Whole-cell lysates were analyzed by 10% SDS-PAGE and Western blotting with the anti-E-Syt1 antibody or anti-E-Syt2 antibody, as indicated. Membranes were further probed with the anti-β-actin antibody for protein loading control. Molecular masses indicated on the right of the blots were estimated using molecular-mass markers run in the same gel. Blots are representative of five separate experiments and were analyzed using FIJI ImageJ 1.54f software. Scatter plots represent E-Syt1 or E-Syt2 protein expression presented as mean ± SEM and expressed as fold increase over the level in the non-tumoral cell line MCF10A. Data were statistically analyzed using the Kruskal–Wallis test with multiple comparisons (Dunn’s test) (* *p* < 0.05 and **** *p* < 0.0001 as compared to MCF10A cells).

Breast cancer cells have been shown to remodel the expression and/or function of SOCE key molecular elements, including Orai channels [22,27]. As E-Syts have been reported to support SOCE by participating in the formation of ER–PM junctions [20], we have investigated the remodeling of the expression and function of E-Syt1 and E-Syt2 in breast cancer cells. First, we have investigated the expression of E-Syt1 and E-Syt2 in non-tumoral and tumoral breast cells. For non-tumoral breast epithelial cells, we studied the MCF10A cell line, and we used two ER+ breast cancer cell lines: MCF7 and T47D, as well as two TNBC cell lines: MDA-MB-231 and BT20 cells. At the transcript level, qRT-PCR analysis revealed a greater expression of E-Syt1 mRNA transcript in both ER+ breast cancer cell lines investigated as compared to non-tumor MCF10A cells (*p* < 0.001). By contrast, E-Syt1 is downregulated in BT20 TNBC cells (Figure 1a; *p* < 0.01). As for E-Syt2, analysis of the expression of E-Syt2 at the transcript level revealed a significantly greater expression in the ER+ breast cancer cell lines MCF7 and T47D (Figure 1a; *p* < 0.0001) and was significantly attenuated in the TNBC cell line MDA-MB-231 as compared to non-tumoral MCF10A cells.

The protein expression of E-Syt1 and E-Syt2 was performed by Western blotting, and data were normalized to the β-actin expression. Our results show an increased expression of E-Syt1 in the TNBC cell line MDA-MB-231 and the ER+ breast cancer cell lines, with predominant expression of E-Syt1 at the protein level in T47D cells (Figure 1b; *p* < 0.05). The expression of E-Syt2 was significantly greater in all the breast cancer cell lines investigated as compared to MCF10A (*p* < 0.05) with a greater expression in BT20 cells (Figure 1b; *p* < 0.0001). 

### 2.2. Role of E-Syt1 and E-Syt2 in Thapsigargin-Induced Ca^2+^ Release and Entry in Breast Cancer and Non-Tumoral Breast Epithelial Cells 

**Figure 2:**
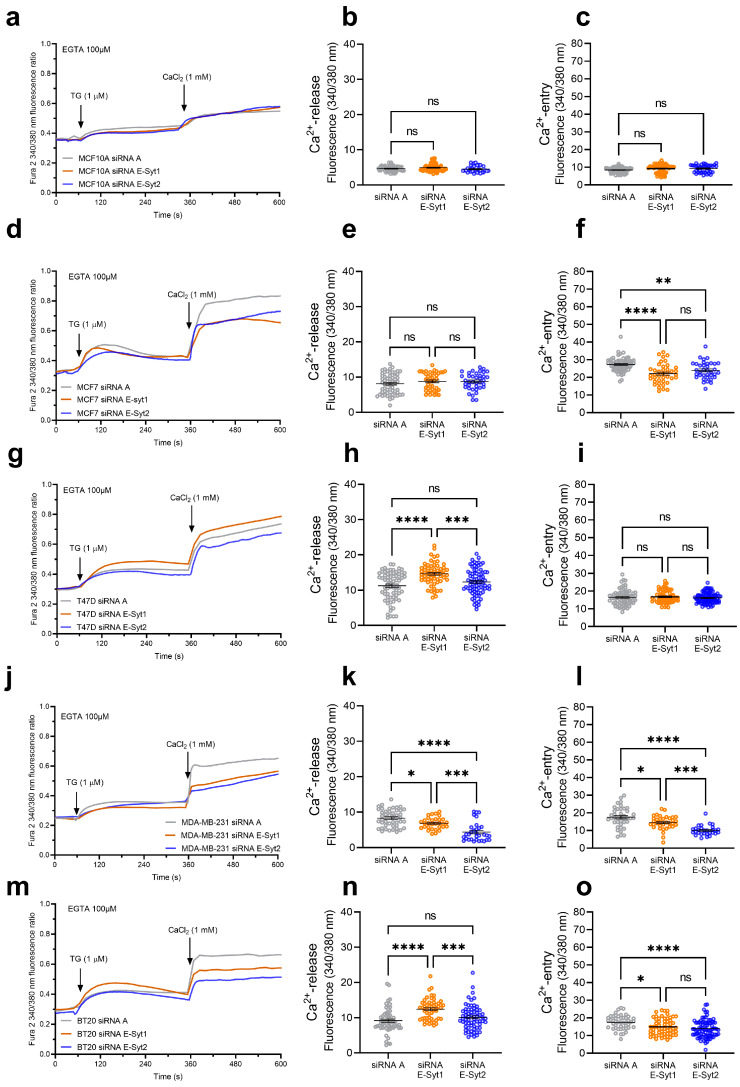
Functional role of E-Syt1 and E-Syt2 in Ca^2+^ release and entry in non-tumoral breast epithelial cells and breast cancer cells. MCF10A (**a**–**c**), MCF7 (**d**–**f**), T47D (**g**–**i**), MDA-MB-231 (**j**–**l**) and BT20 (**m**–**o**) cells were transfected with siRNA E-Syt1, siRNA E-Syt2 or scramble siRNA (siRNA A), as indicated. Seventy-two hours after transfection, cells were loaded with fura-2 and perfused with a Ca^2+^-free medium (100 µM EGTA added). Cells were then stimulated with TG (1 µM) followed by reintroduction of external Ca^2+^ (final concentration 1 mM) to initiate Ca^2+^ entry. Scatter plots represent TG-induced Ca^2+^ release and entry in the cells, expressed as the integral of the increase in the fura-2 fluorescence ratio for 3 and 2½ min, respectively, after the addition of TG or Ca^2+^, as described in Materials and Methods. Data are mean ± SEM of 30–90 cells in 3–5 days. Data were statistically analyzed using the Kruskal–Wallis test combined with Dunn’s post hoc test (ns: not significant, * *p* < 0.05, ** *p* < 0.01, *** *p* < 0.001 and **** *p* < 0.0001).

To investigate the possible functional role of E-Syt1 and E-Syt2 in Ca^2+^ mobilization in breast cancer cells, we transfected cells with siRNA E-Syt1, siRNA E-Syt2 or scramble siRNA as control. Non-tumoral breast epithelial MCF10A cells, ER+ breast cancer MCF7 and T47D cells and TNBC breast cancer MDA-MB-231 and BT20 cells were transfected with siRNA E-Syt-1, siRNA E-Syt2 or scramble siRNA (siRNA A) as control. Seventy-two hours after transfection, the expression of E-Syt1 and E-Syt2 in cells transfected with the corresponding siRNA was significantly reduced (Figures S1 and S2; *p* < 0.05). In the absence of extracellular Ca^2+^, cell treatment with 1 µM thapsigargin (TG), an inhibitor of the sarco/endoplasmic reticulum Ca^2+^-ATPase (SERCA), induced a transient (or sustained, depending on the cell type) increase in the fura-2 fluorescence ratio mediated by Ca^2+^ release from the ER. Subsequent addition of Ca^2+^ to the extracellular medium leads to a rise in the fura-2 fluorescence ratio that is indicative of Ca^2+^ influx (Figure 2). E-Syt1 expression silencing did not alter TG-induced Ca^2+^ release or entry in non-tumoral MCF10A cells, and similar results were observed when E-Syt2 expression was attenuated (Figure 2a–c). In ER+ breast cancer MCF7 cells, E-Syt1 or E-Syt2 knockdown was without effect on TG-induced Ca^2+^ release from the internal stores but significantly attenuated Ca^2+^ entry, thus indicating that both proteins are required for the activation of SOCE in this cell line (Figure 2d–f; *p* < 0.01). In the ER+ T47D cell line, attenuation of E-Syt1 expression enhanced TG-evoked Ca^2+^ release from the ER without having any significant effect on SOCE (Figure 2g–i; *p* < 0.001). Meanwhile, silencing E-Syt2 was without effect on Ca^2+^ release or entry mediated by TG in T47D cells (Figure 2g–i). Concerning TNBC cell lines, the knockdown of both E-Syts significantly attenuated TG-evoked SOCE, and the effect of this experimental maneuver was found to be cell-specific (Figure 2j–o; *p* < 0.05). These findings indicate that E-Syt1 and E-Syt2 play a relevant role in SOCE in TNBC breast cancer cells and the ER+ breast cancer MCF7 cells.

### 2.3. Functional Role of E-Syt1 and E-Syt2 in Breast Cancer and Non-Tumoral Breast Epithelial Cell Migration

**Figure 3:**
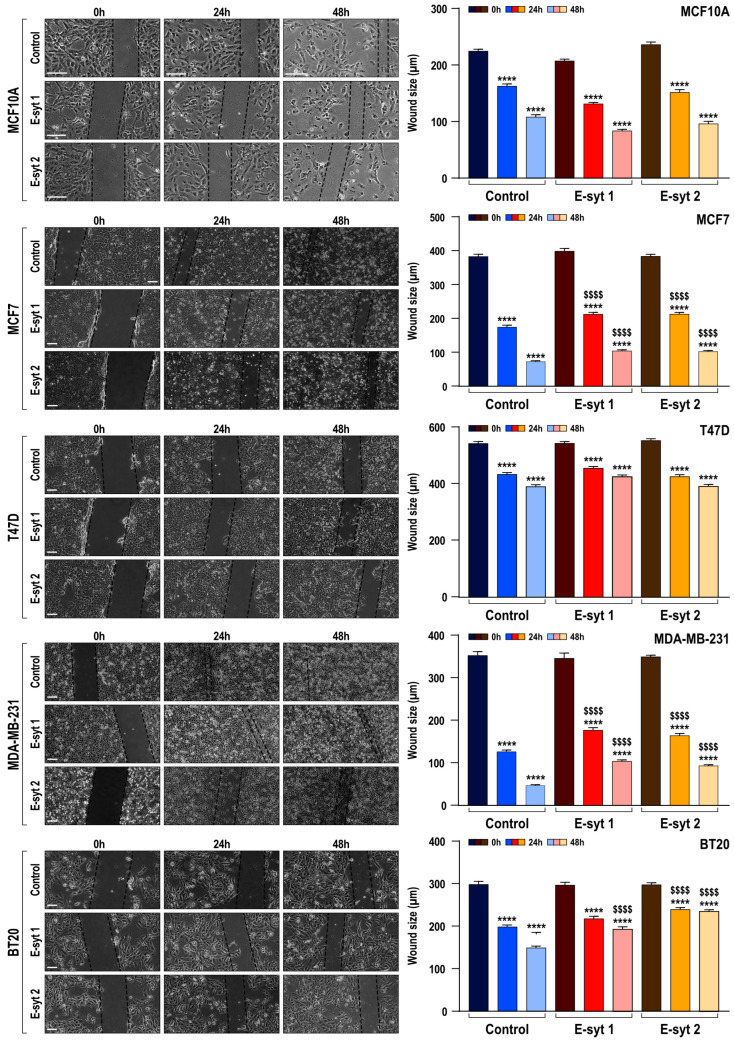
Effect of knockdown of E-Syt1 and E-Syt2 on migration of non-tumoral breast epithelial cells and breast cancer cells. Non-tumoral breast epithelial MCF10A cells, ER+ breast cancer MCF7 and T47D cells and TNBC MDA-MB-231 and BT20 cells were transfected with siRNA E-Syt1, siRNA E-Syt2 or scramble siRNA (siRNA A), as indicated. Seventy-two hours after transfection cells were subjected to the wound healing assay. Bar graphs represent the quantification of the wound size, in micrometers, under different conditions. Data are presented as mean  ±  standard error of the mean (SEM) and were statistically analyzed using the Kruskal–Wallis test with multiple comparisons (Dunn’s test). **** *p*  <  0.0001 as compared to t = 0. ^$$$$^ *p*  <  0.0001 as compared to the wound size at the corresponding time in the cells transfected with scramble siRNA. Images were acquired at 0, 24 and 48 h from the beginning of the assay. Images are representative of six independent experiments (a total of 90–120 measurements) for each experimental condition. The dotted lines define the areas lacking cells. Scale bar: 200 μm.

Our results have provided evidence for the relevant role of E-Syt1 and E-Syt2 in the activation of SOCE. Hence, we have further investigated the functional role of these proteins in cell migration, a hallmark of cancer cells. Analysis of cell migration in non-tumoral MCF10A cells, as well as ER+ MCF7 and T47D and TNBC MDA-MB-231 and BT20 cells, was performed as described in Materials and Methods. As shown in Figure 3, untreated cells significantly reduced the wound size during the first 48 h (*p*  <  0.0001; *n*  =  6). Silencing E-Syt1 expression significantly attenuated the migration of MCF-7 and MDA-MB-231 cells both after 24 and 48 h (Figure 3; *p*  <  0.0001; *n*  =  6). Furthermore, E-Syt1 knockdown resulted in a significant inhibition of BT20 cell migration after 48 h (Figure 3; *p*  <  0.0001; *n*  =  6). By contrast, E-Syt1 expression attenuation was without significant effect on MCF10A and T47D cell migration, which is consistent with the effect observed in SOCE in these cells. Similar results were observed when the expression of E-Syt2 was reduced. E-Syt2 knockdown significantly attenuated MCF7, MDA-MB-231 and BT20 cell migration both at 24 and 48 h, without having any significant effect on cell migration in MCF10A and T47D. These findings show interesting parallelism with the functional role of E-Syt proteins on SOCE in non-tumoral and breast cancer cells and strongly suggest a role for SOCE in cell migration.

### 2.4. Functional Role of E-Syt1 in Cell Viability in ER+ Breast Cancer and Non-Tumoral Breast Epithelial Cells

**Figure 4:**
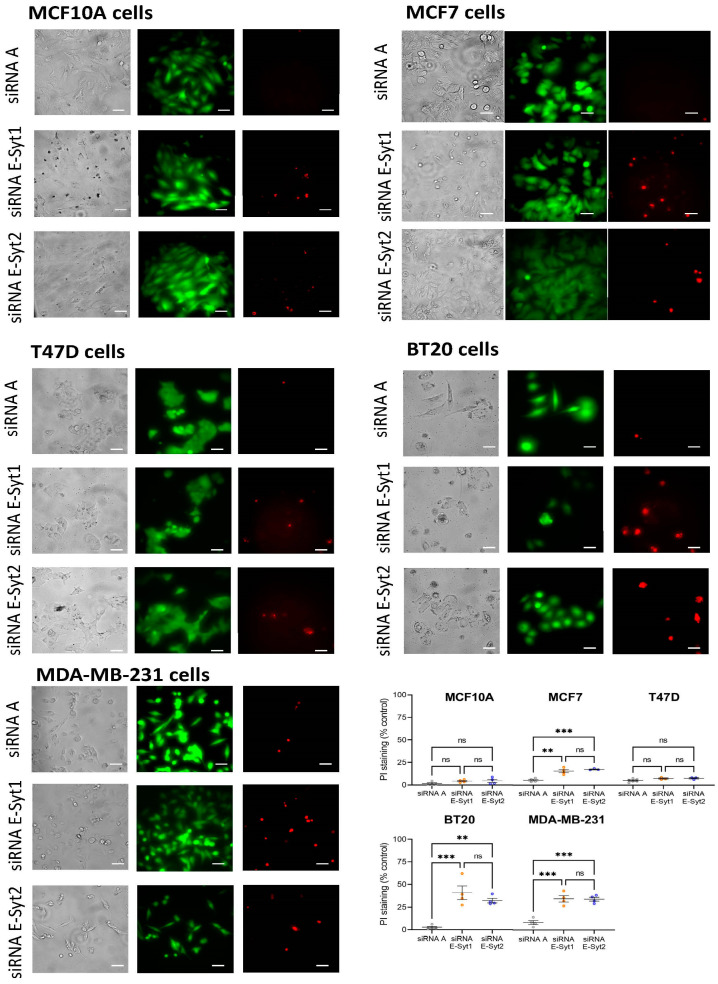
Effect of E-Syt1 and E-Syt2 knockdown on cell viability. Non-tumoral breast epithelial MCF10A cells, ER+ breast cancer MCF7 and T47D cells and TNBC MDA-MB-231 and BT20 cells were transfected with siRNA E-Syt1, siRNA E-Syt2 or scramble siRNA (siRNA A), as indicated. Seventy-two hours after transfection cells were loaded with calcein and propidium iodide (PI). Cell staining was visualized using an EVOS FL Auto 2 cell imaging system as described in Materials and Methods. Scatter plots represent PI staining, normalized to calcein staining, under the different conditions, presented as % of control cells (cells transfected with siRNA A) and are expressed as mean ± SEM. The images shown are representative of 2–5 independent experiments. Analysis of statistical significance was performed using the Kruskal–Wallis test with multiple comparisons (Dunn’s test). ns: not significant, ** *p* < 0.01 and *** *p* < 0.001. Scale bar: 25 µm.

As E-Syt1 and E-Syt2 are required for full activation of SOCE in a variety of breast cancer cells, including ER+ breast cancer MCF7 cells and the TNBC cell lines MDA-MB-231 and BT20, we have explored their roles in cell viability by using the cell-permeant dye calcein and propidium iodide (PI). Cells were transfected with siRNA E-Syt1, siRNA E-Syt2 or scramble siRNA (siRNA A) as control, and 72 h later, calcein and propidium iodide fluorescence was assessed. Our results indicate that most cells were viable after transfection with siRNA A. E-Syt1 knockdown significantly enhanced PI staining in MCF7, MDA-MB-231 and BT20 cells (Figure 4; *p* < 0.001); while PI staining of non-tumoral MCF10A and ER+ breast cancer T47D cells was unaffected by transfection of siRNA E-Syt1 (Figure 4). Similar results were obtained by silencing the expression of E-Syt2 (Figure 4; *p* < 0.001). These findings indicate that E-Syt1 and E-Syt2 knockdown attenuate cell viability in ER+ breast cancer MCF7 and TNBC MDA-MB-231 and BT20 cells without having any effect on non-tumoral cells or the ER+ tumoral cell line T47D. These observations are consistent with the functional role of E-Syt1 and E-Syt2 in the activation of SOCE.

## 3. Discussion

Mammalian extended synaptotagmins (E-Syts) are ER-resident proteins that tether the ER and PM facilitating membrane contact sites that play relevant roles in different cellular functions, including lipid transfer, metabolism and calcium signaling [15]. Among the mechanisms that occur at sites of close apposition between the ER and PM stands SOCE, an almost universal mechanism for Ca^2+^ influx regulated by the filling state of the ER Ca^2+^ store [28]. SOCE core proteins, the ER Ca^2+^ sensor STIM1 and the CRAC channel subunit Orai1, have been associated with cancer progression and metastasis [29,30,31]. In breast cancer cells, impairment of Orai1 function or expression using dominant negative mutants or specific RNAi attenuates proliferation and migration, arrests cell cycle progression and reduces the viability and the resistance to chemotherapeutic agents as well as self-renewal of breast cancer stem cells [27,32,33,34,35]. It has been reported that the interaction between Orai1 channels and STIM isoforms is supported by E-Syt proteins. The short E-Syt2 isoform recruits STIM1 at ER–PM junctions [18]. Furthermore, E-Syt2 and E-Syt3 support the interaction between STIM2 and Orai1 at ER–PM contact sites that modulate Ca^2+^ influx at resting conditions [19], and finally, E-Syt1 is essential for the formation of ER–PM junctions that support Orai1–STIM1 interaction [20]. Here we show that ER+ breast cancer cell lines MCF7 and T47D overexpress E-Syt1 both at the transcript and protein levels. On the other hand, the TNBC cell lines MDA-MB-231 and BT20 exhibit similar or even reduced E-Syt expression at the transcript level as compared to MCF10A non-tumoral cells. Interestingly, at the protein level, the ER+ breast cancer cell lines investigated as well as the TNBC MDA-MB-231 cell line show enhanced E-Syt1 expression, and all the ER+ and TNBC cell lines investigated show E-Syt2 overexpression. Interestingly, our results indicate that the knockdown of E-Syt1 or E-Syt2 significantly inhibits SOCE in ER+ MCF7 cells and TNBC MDA-MB-231 and BT20 cells. By contrast, E-Syt1 and E-Syt2 expression silencing did not significantly alter SOCE in the T47D cell line or the non-tumoral breast epithelial cell line MCF10A used as control. 

The reason for the different relevance of E-Syt1 in SOCE in MCF7 and T47D cells is unclear. MCF7 and T47D are among the most used ER+ breast cancer cell lines and are widely used as experimental cellular models for breast cancer studies. Both cell lines derive from a metastatic site of pleural effusion and express estrogen and progesterone receptors [36]. Nevertheless, several differences have been reported between these cell lines. Among them, T47D and MCF7 exhibit differential expression of many proteins. For instance, T47D cells exhibit a higher expression of several proteins associated with cell growth, apoptosis resistance and carcinogenesis; by contrast, other proteins involved in transcription repression and apoptosis are less abundant in T47D than in MCF7 [37]. Furthermore, both cell lines exhibit differences in cell bioenergetic functions, with MCF7 being less tolerant to cellular stress [38]. The mechanism underlying the activation of apoptosis in both cell types has also been reported to be different [39]. Considering these observations, it would not be surprising to find differences in the mechanism modulating SOCE in both cell types.

Our results also provide evidence for the role of E-Syt1 and E-Syt2 in cell migration and viability in ER+ MCF7 cells and TNBC MDA-MB-231 and BT20 cells, while these proteins are not required for MCF10A or T47D cell migration and survival. These findings are consistent with a previously reported role for SOCE in the development of cancer hallmarks [40,41] as well as with the requirement of E-Syt1 and E-Syt2 for the activation of SOCE in MCF7 and TNBC cells. Nevertheless, we cannot rule out that E-Syt1 and E-Syt2 participate in cell migration and viability by mechanisms different from Ca^2+^ signaling, as membrane contact sites have been reported to be essential for cancer progression [42], but attenuation of SOCE in breast cancer cells has been reported to have significant effects on cell viability and cell cycle progression [27,33,43]. To the best of our knowledge, this might be the first description of a role for E-Syt1 and E-Syt2 in cell viability, as previous studies in mice have revealed that inactivation of all three E-Syt genes has no detectable effect on mouse viability [44], although embryonic fibroblasts from E-Syt2^−/−^ and E-Syt3^−/−^ mice exhibit reduced survival under oxidative stress [45]. Altogether, our findings provide evidence for the functional role of E-Syt1 and E-Syt2 in the activation of SOCE and subsequent migration and viability of breast cancer cells.

## 4. Materials and Methods

### 4.1. Materials and Reagents 

Fura-2 acetoxymethyl ester (fura-2/AM) was purchased from Molecular Probes (Leiden, The Netherlands). Thapsigargin (TG), rabbit polyclonal anti-β-actin antibody (catalog number A2066, epitope: amino acids 365–375 of human β-actin), insulin, epidermal growth factor, bovine serum albumin (BSA), HEPES (4-(2-hydroxyethyl)piperazine-1-ethanesulfonic acid), EGTA (ethylene glycol-bis(2-aminoethylether)-N,N,N′,N′-tetraacetic acid) and EDTA (ethylenedinitrilotetraacetic acid) were obtained from MilliporeSigma (Burlington, MA, USA). Trypsin, fetal bovine serum (FBS), horse serum, hydrocortisone, penicillin/streptomycin, BCA protein assay kit, Live/Dead viability/cytotoxicity kit, high-glucose Dulbecco’s modified Eagle’s medium (DMEM), rabbit polyclonal anti-E-Syt1 antibody (catalog number 50-156-4938: epitope: aa1054-aa1104) and rabbit polyclonal anti-E-Syt2 antibody (catalog number PA5-51689, epitope: aa489-aa618) were purchased from ThermoFisher Scientific (Waltham, MA, USA). SYBR^®^ Premix Ex Taq™ (Takara Bio Inc., Otsu, Shiga, Japan). DharmaFECT kb was from Horizon Discovery (Horizon Discovery (Waterbeach, UK)). Horseradish peroxidase-conjugated goat anti-mouse IgG and goat anti-rabbit IgG antibodies were from Jackson laboratories (West Grove, PA, USA). Enhanced chemiluminescence detection reagents were obtained from Pierce (Cheshire, UK). The fluorescent goat anti-rabbit IgG StarBright Blue 700 antibody was from Bio-Rad (Bio-Rad Laboratories Inc., Hercules, CA, USA). siRNA E-Syt1 (3 target specific 19–25nt siRNA; catalog number sc-95714) and siRNA E-Syt2 (5 target specific 19–25 nt siRNA; catalog number sc-89470) were purchased from Santa Cruz (Dallas, TX, USA). All other reagents were of analytical grade.

### 4.2. Cell Culture and Transfections

ER+ MCF7 and T47D breast cancer cell lines and triple-negative MDA-MB-231 and BT20 breast cancer cell lines were obtained from ATCC (Manassas, VA, USA) and cultured at 37 °C with a 5% CO_2_ in high-glucose DMEM supplemented with 10% (*v*/*v*) FBS and 100 U/mL penicillin and streptomycin, as described previously [23]. The MCF10A cell line was from ATCC. MCF10A cells were cultured at 37 °C with a 5% CO_2_ in DMEM-F12, supplemented with 5% (*v*/*v*) horse serum, 0.5 μg/mL hydrocortisone, 10 μg/mL insulin, 20 ng/mL epidermal growth factor and 100 ng/mL cholera toxin.

For transient transfections, cells with 60% to 80% confluency were transfected with the indicated siRNAs using DharmaFECT kb transfection reagent. Cells were used 72 h after transfection. For Western blotting, cells (at a 2 × 10^6^ concentration) were plated in a 100 mm petri dish, while, for Ca^2+^ imaging and viability, assay cells (with a 4 × 10^5^ concentration) were seeded in a 35 mm six-well multi-dish.

### 4.3. Determination of Cytosolic Free-Ca^2+^ Concentration

Fura 2 loading was obtained by cell incubation with 2 µM fura 2/AM for 30 min at 37 °C. Cultured cells on coverslips mounted on a perfusion chamber were placed on the stage of an epifluorescence inverted microscope (Nikon Eclipse Ti2, Amsterdam, The Netherlands), and images were acquired and analyzed using the software NIS-Elements Imaging Software v.5.02.00 (Nikon, Amsterdam, The Netherlands). Cells were superfused with HEPES-buffered saline containing 125 mM NaCl, 5 mM KCl, 1 mM MgCl_2_, 5 mM glucose, 25 mM HEPES and pH 7.4, supplemented with 0.1% (*w*/*v*) BSA. Samples were alternatively excited at 340/380 nm using a high-speed monochromator (Optoscan ELE 450, Cairn Research, Faversham, UK). Fluorescence emission of the samples was recorded at 505 nm using a sCMOS camera (PCO Panda 4.2 (Excelitas PCO GmbH, Kelheim, Germany)). Fluorescence ratio (F340/F380) was calculated pixel by pixel. TG-evoked Ca^2+^ release and SOCE were measured as the integral of the rise in the fura-2 fluorescence ratio for 3 and 2½ min, respectively, after the addition of TG (for Ca^2+^ release) or Ca^2+^ (for SOCE), respectively.

### 4.4. Western Blotting

Samples of cell lysates were resolved by 10% SDS-PAGE, and proteins were electrophoretically transferred onto nitrocellulose membranes for subsequent probing. Blots were incubated with 10% (*w*/*v*) BSA in Tris-buffered saline with 0.1% Tween 20 (TBST) overnight to block residual protein-binding sites. Detection of E-Syt1, E-Syt2 and β-actin was carried out by incubation with the anti-E-Syt1 or anti-E-Syt2 antibodies for 1 h and diluted 1:1000 or 1:1500 in TBST, respectively, or by incubation with the anti-β-actin antibody diluted 1:2000 in TBST for 1 h. To detect the primary antibody, blots were incubated for 1 h with fluorescent goat anti-rabbit IgG StarBright Blue 700 antibody or goat anti-mouse IgG StarBright Blue 700 diluted 1:3000 in TBST. Additionally, primary antibodies were also detected using horseradish peroxidase-conjugated goat anti-rabbit IgG antibody or horseradish peroxidase-conjugated goat anti-mouse IgG antibody diluted 1:10,000 in TBST and then, in this case, blots were exposed to enhanced chemiluminiscence reagents for 5 min. In both cases, the antibody binding was detected with a ChemiDoc MP Imaging System (Bio-Rad Laboratories, Inc., Hercules, CA, USA) and the density of bands was measured using Fiji-ImageJ 1.54f software (NIH, Bethesda, MD, USA).

### 4.5. Wound Healing Assay 

Non-tumoral MCF10A cells, ER+ breast cancer MCF7 and T47D cells and TNBC MDA-MB-231 and BT20 cells were seeded in a 35 mm six-well multi-dish to obtain confluence after 24 h. Next, cells were cultured in a medium supplemented with 1% serum, and a wound was created using a sterile 200 μL plastic pipette tip. Images were taken immediately or at the times indicated using an EVOS FL Auto 2 cell imaging system (ThermoFisher Scientific (Waltham, MA, USA). Cell migration was quantitated using fiji ImageJ (NIH, Bethesda, MD, USA). Between 15 and 20 measurements were randomly taken along the wound for every single experiment.

### 4.6. Determination of Cell Viability 

Cell viability was tested using the Live/Dead viability/cytotoxicity kit. Briefly, cells were incubated with calcein-AM and propidium iodide (PI) following the manufacturer’s instructions, and samples were excited at 430 nm and 555 nm for calcein and propidium iodide (PI), respectively. Fluorescence emission at 542 nm (for viable cells) and 624 nm (for dead cells) was recorded using an EVOS FL Auto 2 cell imaging system (ThermoFisher Scientific (Waltham, MA, USA). Cell viability was quantitated using Fiji ImageJ 1.54f software (NIH, Bethesda, MD, USA).

### 4.7. Quantitative RT-PCR

Total RNA isolation and single-strand cDNA synthesis were performed in culture cells. The primers used are depicted in Table 1. SYBR green qRT-PCR was performed using SYBR^®^ Premix Ex Taq™ (Takara Bio Inc., Otsu, Shiga, Japan) in an Applied Biosystems STEPONE Real-Time thermal cycler (Life Technologies Corporation, Carlsbad, CA, USA) as described previously. PCR products were obtained using the following cycling conditions: 96 °C for 2 min, followed by 35 cycles of 96 °C for 15 s, 48–56 °C for 25 s and finished with 72 °C for 10 min. mRNA abundance was calculated by the comparative CT (ΔΔCT) method using the equation: RQ = 2^−ΔΔCT^. The amount of mRNA transcripts was normalized to GAPDH expression and represented as mean expression relative to MCF10A specific mRNA ± S.E.M.

### 4.8. Statistical Analysis

Statistical analysis was performed using the Kruskal–Wallis test combined with Dunn’s post hoc test (GraphPad Prism Windows v.8, San Diego, CA, USA). For comparison between the two groups, the Mann–Whitney U test (or Student’s *t* test for the statistical analysis of Ca^2+^ measurements) was used. A *p*-value < 0.05 was considered statistically significant.

## 5. Conclusions

Our results indicate that E-Syt1 and E-Syt2 are overexpressed in most breast cancer cell lines investigated at the protein level, as compared to non-tumoral MCF10A breast epithelial cells. E-Syt1 and E-Syt2 are required for full activation of SOCE in ER+ breast cancer MCF7 cells and the TNBC cell lines MDA-MB-231 and BT20, most likely by supporting the close apposition of Orai1 and STIM1 in ER–PM junctions. Furthermore, both proteins play a functional role in cell migration and viability in the above-mentioned cell lines, thus suggesting that these proteins play an important role in breast cancer cell biology.

## Figures and Tables

**Table 1:** Primers used in qRT-PCR.

Protein	Forward Primer	Reverse Primer
E-Syt1	TCGCAAGACTAGGCAACCTC	CCAAATACACAGGTATCAGCACCA
E-Syt2	CCTGAGAAAGACAGTGACAGGAAG	GCCCAGCCTACTTTAACTGCT
E-Syt3	CTCGAGCTTGGGAGACAGATG	CCCAGGTAGCCAGCTAGGTA
GAPDH	GTCTCCTCTGACTTCAACAGCG	ACCACCCTGTTGCTGTAGCCAA

## Data Availability

The data presented in this study are available on request from the corresponding author.

## Supplementary Materials

The following supporting information can be downloaded at: https://www.mdpi.com/article/10.3390/cancers16142518/s1, Figure S1. E-Syt1 expression knockdown in MCF10A, MCF7, T47D, MDA-MB-231 and BT20 cells. Figure S2. E-Syt2 expression knockdown in MCF10A, MCF7, T47D, MDA-MB-231 and BT20 cells. Figure S3. Uncropped blots of Figure 1b. Figure S4. Uncropped blots of Figure S1a–e. Figure S5. Uncropped blots of Figure S2a–e.
