# Supplementary material for: Extended Synaptotagmins 1 and 2 Are Required for Store-Operated Calcium Entry, Cell Migration and Viability in Breast Cancer Cells
Source: Cancers (Basel). 2024 Jul 11;16(14):2518. doi: 10.3390/cancers16142518 (PMC11274662; doi:10.3390/cancers16142518)
Supplement: Supplementary file 1 [file cancers-16-02518-s001.zip › cancers-3072064-supplementary.pdf]

Supplementary materials

Extended Synaptotagmins 1 and 2 Are Required for Store-Operated Calcium Entry, Cell Migration and Viability in Breast Cancer Cells

Pedro C. Redondo [1†*], Jose J. Lopez [1†], Sandra Alvarado [1], Isaac Jardin [1], Joel Nieto-Felipe, Alvaro Macias-Diaz, Vanesa Jimenez-Velarde, Gines M. Salido [1] and Juan A. Rosado [1*]

Figures S1, S2, S3, S4 and S5

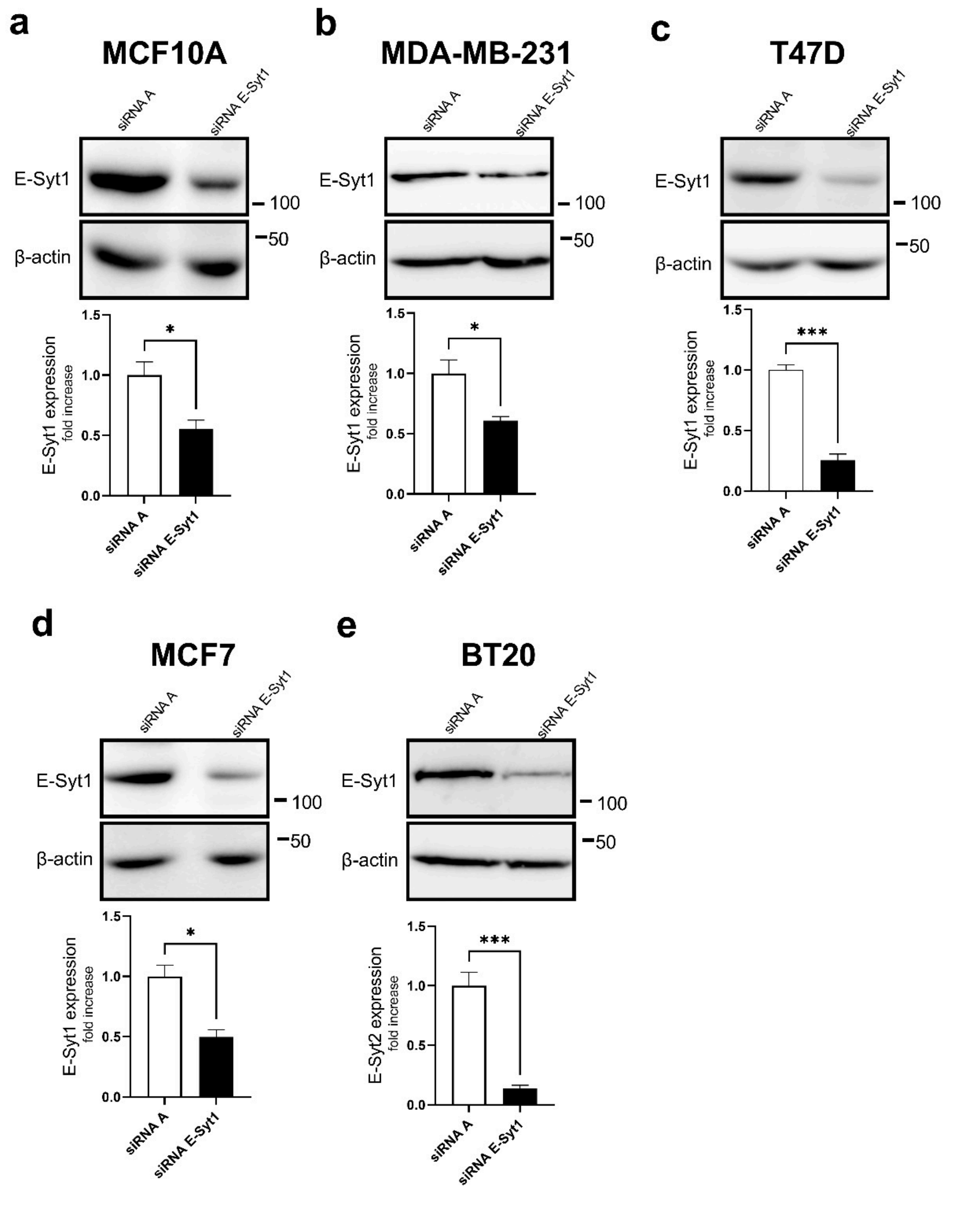

Figure S1. E-Syt1 expression knockdown in MCF10A, MCF7, T47D, MDA-MB-231 and BT20 cells. MCF10A, MCF7, T47D, MDA-MB-231 and BT20 cells were transfected with siRNA E-Syt1 or scramble siRNA (siRNA A), as indicated. After 72 h, cells were lysed and analyzed using Western blotting with the anti-E-Syt1 antibody, followed by reprobing with the anti-β-actin antibody for protein loading control. Molecular masses indicated on the right were determined using molecular-mass markers run in the same gel. Blots are representative of three separate experiments. Bar graphs represent E-Syt1 expression under the different experimental conditions. Data are presented as mean ± SEM of three independent experiments and expressed as a percentage of control (cells transfected with siRNA A). Data were statistically analyzed using the Mann–Whitney U-test. * $p < 0.05$ and *** $p < 0.001$.

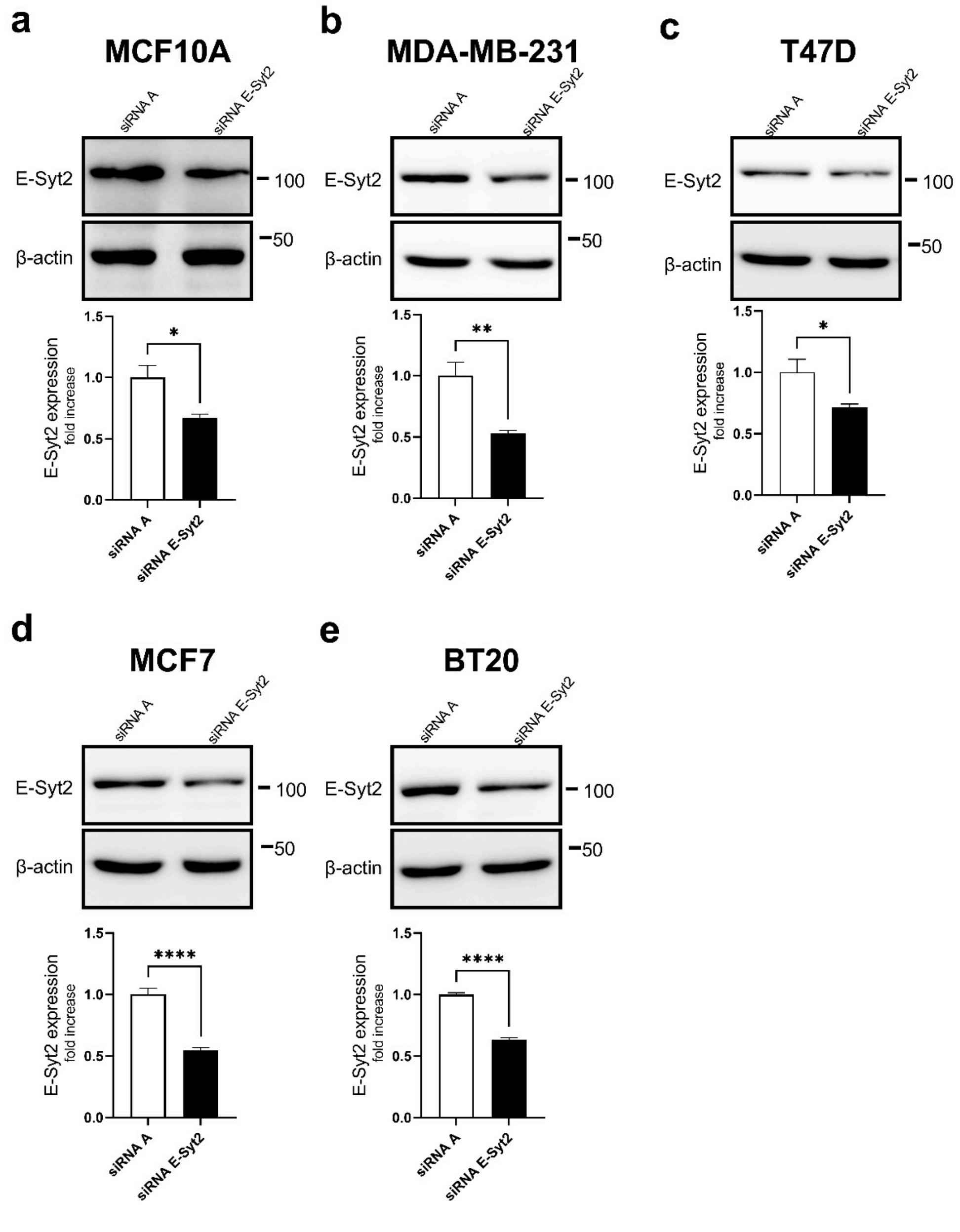

Figure S2. E-Syt2 expression knockdown in MCF10A, MCF7, T47D, MDA-MB-231 and BT20 cells. MCF10A, MCF7, T47D, MDA-MB-231 and BT20 cells were transfected with siRNA E-Syt2 or scramble siRNA (siRNA A), as indicated. After 72 h, cells were lysed and analyzed using Western blotting with the anti-E-Syt2 antibody, followed by reprobing with the anti-β-actin antibody for protein loading control. Molecular masses indicated on the right were determined using molecular-mass markers run in the same gel. Blots are representative of three to four separate experiments. Bar graphs represent E-Syt2 expression under the different experimental conditions. Data are presented as mean ± SEM of three independent experiments and expressed as a percentage of control (cells transfected with siRNA A). Data were statistically analyzed using the Mann–Whitney U-test. * $p<0.05$, ** $p<0.01$ and **** $p<0.0001$.

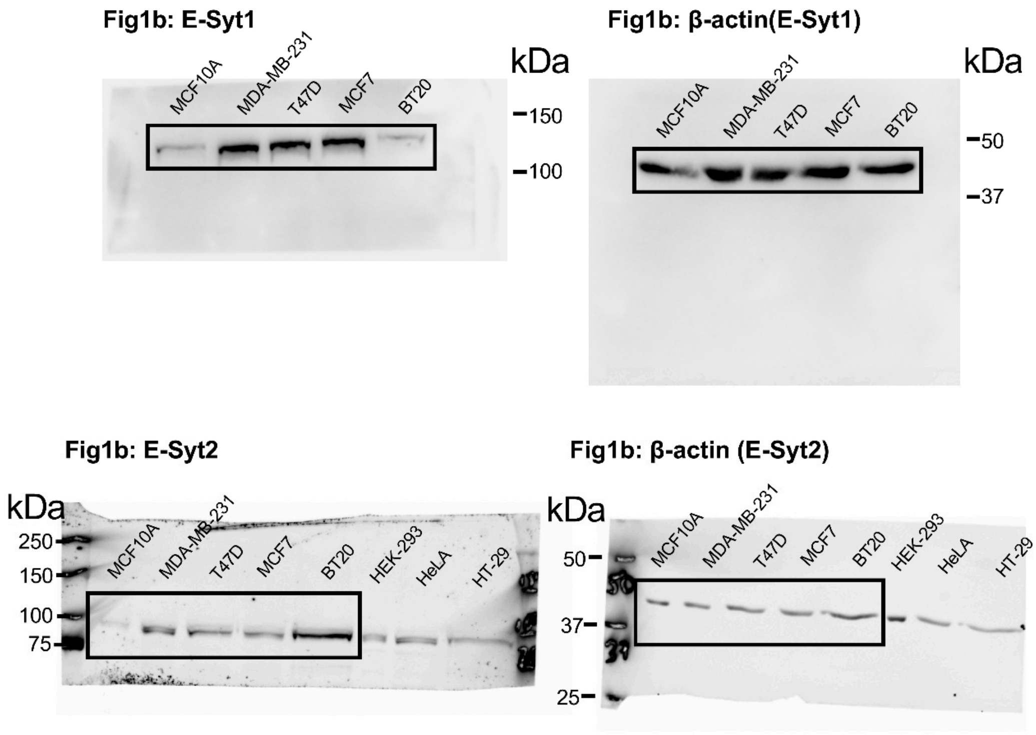

Figure S3. Uncropped blots of Figure 1b.

FigS1a: MCF10A E-Syt1

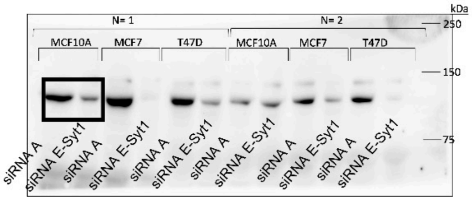

FigS1a: MCF10A β-actin

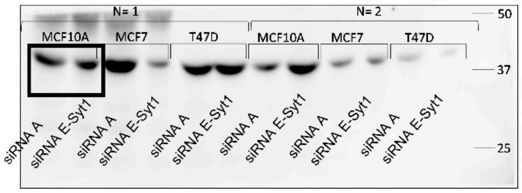

FigS1b: MDA-MB-231 E-Syt1

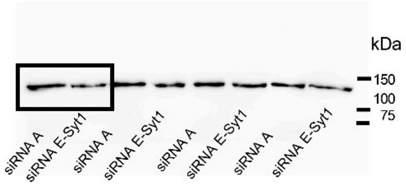

FigS1b: MDA-MB-231 β-actin

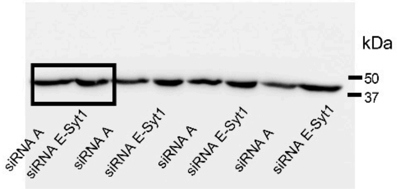

FigS1c: T47D E-Syt1

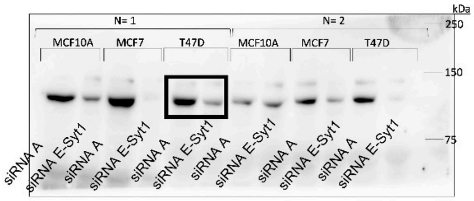

FigS1c: T47D β-actin

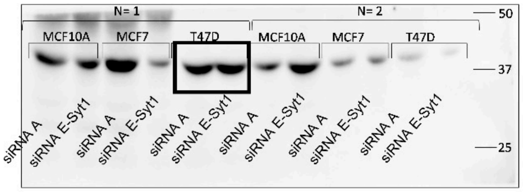

FigS1d: MCF7 E-Syt1

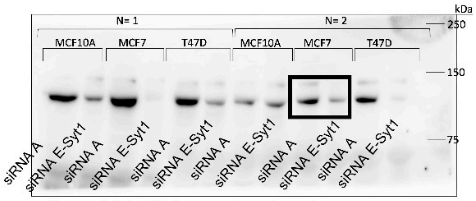

FigS1d: MCF7 β-actin

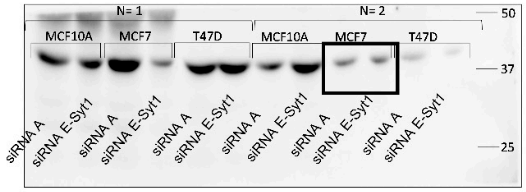

FigS1e: BT20 E-Syt1

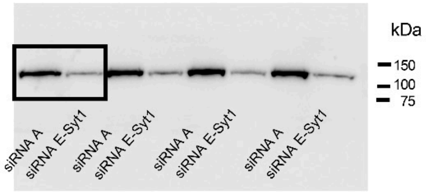

FigS1e: BT20 β-actin

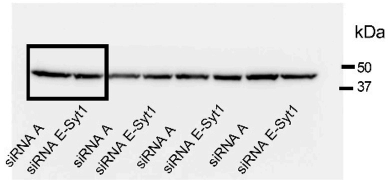

Figure S4. Uncropped blots of Figure S1 (a-e).

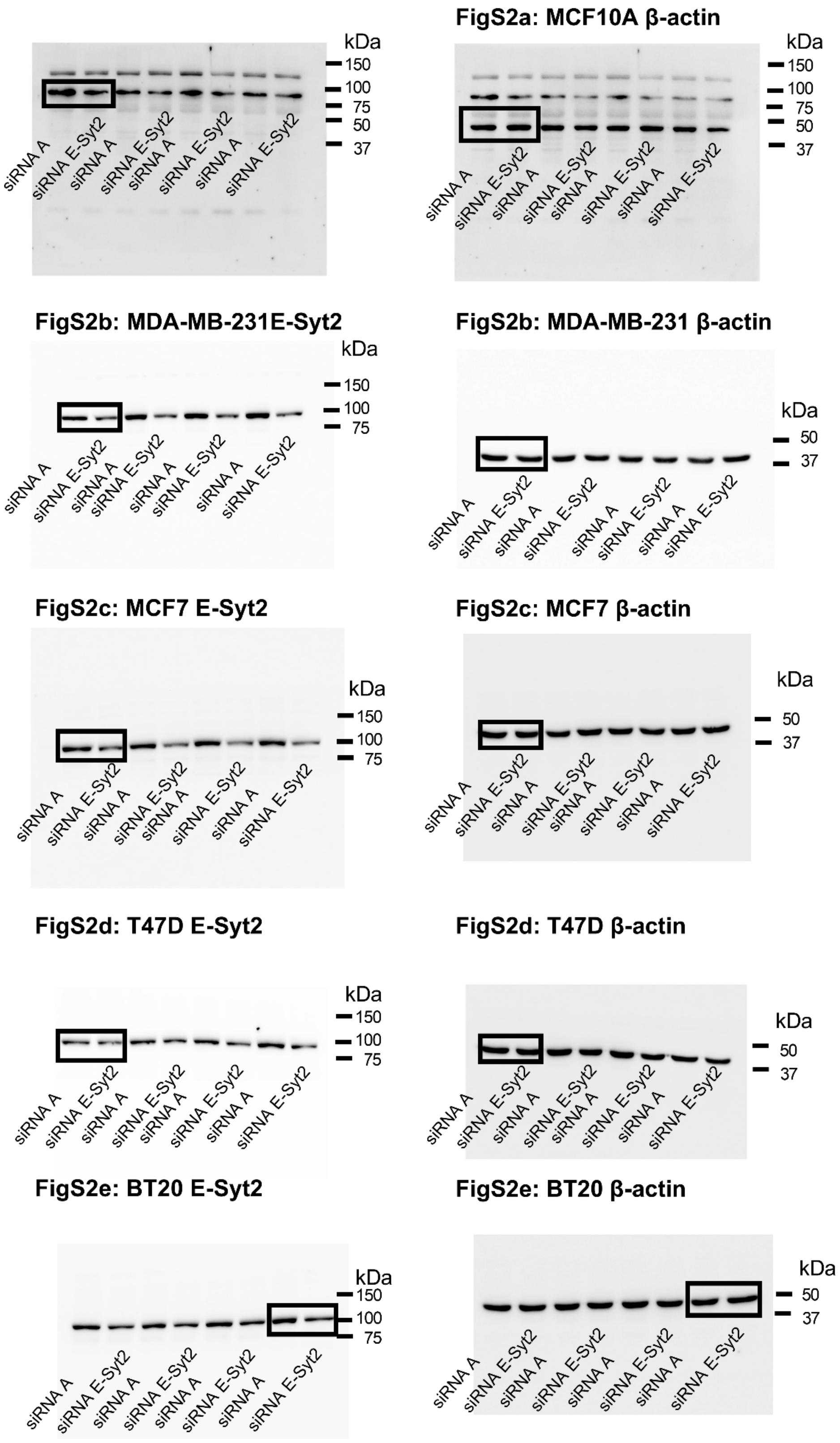

Figure S5. Uncropped blots of Figure S2 (a-e).
